# Novel Class of Viral Ankyrin Proteins Targeting the Host E3 Ubiquitin Ligase Cullin-2

**DOI:** 10.1128/JVI.01374-18

**Published:** 2018-11-12

**Authors:** Valerie Odon, Iliana Georgana, Joe Holley, Jordi Morata, Carlos Maluquer de Motes

**Affiliations:** a Department of Microbial Sciences, University of Surrey, Guildford, United Kingdom; b Centre for Research in Agricultural Genomics, Barcelona, Catalonia, Spain; University of Illinois at Urbana Champaign

**Keywords:** Ankyrin proteins, Cullin ubiquitin system, immune evasion, poxvirus

## Abstract

Ankyrin repeat (ANK) domains are among the most abundant motifs in eukaryotic proteins. ANK proteins are rare amongst viruses, with the exception of poxviruses, which presumably acquired them from the host via horizontal gene transfer. The architecture of poxvirus ANK proteins is, however, different from that of their cellular counterparts, and this precludes a direct acquisition event. Here we combine bioinformatics analysis and quantitative proteomics to discover a new class of viral ANK proteins with a domain organization that relates to cellular ANK proteins. These noncanonical viral ANK proteins, termed ANK/BC, interact with host Cullin-2 via a C-terminal BC box resembling that of cellular Cullin-2 substrate adaptors such as the von Hippel-Lindau protein. Mutagenesis of the BC box-like sequence abrogates binding to Cullin-2, whereas fusion of this motif to an ANK-only protein confers Cullin-2 association. We demonstrated that these viral ANK/BC proteins are potent immunomodulatory proteins suppressing the activation of the proinflammatory transcription factors NF-κB and interferon (IFN)-responsive factor 3 (IRF-3) and the production of cytokines and chemokines, including interferon, and that association with Cullin-2 is required for optimal inhibitory activity. ANK/BC proteins exist in several orthopoxviruses and cluster into 2 closely related orthologue groups in a phylogenetic lineage that is separate from that of canonical ANK/F-box proteins. Given the existence of cellular proteins with similar architecture, viral ANK/BC proteins may be closely related to the original ANK gene acquired by an ancestral orthopoxvirus. These findings uncover a novel viral strategy to antagonize innate immunity and shed light on the origin of the poxviral ANK protein family.

**IMPORTANCE** Viruses encode multiple proteins aimed at modulating cellular homeostasis and antagonizing the host antiviral response. Most of these genes were originally acquired from the host and subsequently adapted to benefit the virus. ANK proteins are common in eukaryotes but are unusual amongst viruses, with the exception of poxviruses, where they represent one of the largest protein families. We report here the existence of a new class of viral ANK proteins, termed ANK/BC, that provide new insights into the origin of poxvirus ANK proteins. ANK/BC proteins target the host E3 ubiquitin ligase Cullin-2 via a C-terminal BC box domain and are potent suppressors of the production of inflammatory cytokines, including interferon. The existence of cellular ANK proteins whose architecture is similar suggests the acquisition of a host ANK/BC gene by an ancestral orthopoxvirus and its subsequent duplication and adaptation to widen the repertoire of immune evasion strategies.

## Abstract (precis)

Viruses encode multiple proteins aimed at modulating cellular homeostasis and antagonizing the host antiviral response. Most of these genes were originally acquired from the host and subsequently adapted to benefit the virus. ANK proteins are common in eukaryotes but are unusual amongst viruses, with the exception of poxviruses, where they represent one of the largest protein families. We report here the existence of a new class of viral ANK proteins, termed ANK/BC, that provide new insights into the origin of poxvirus ANK proteins. ANK/BC proteins target the host E3 ubiquitin ligase Cullin-2 via a C-terminal BC box domain and are potent suppressors of the production of inflammatory cytokines, including interferon. The existence of cellular ANK proteins whose architecture is similar suggests the acquisition of a host ANK/BC gene by an ancestral orthopoxvirus and its subsequent duplication and adaptation to widen the repertoire of immune evasion strategies.

## INTRODUCTION

The Ankyrin repeat (ANK) motif is a 33-amino-acid sequence that mediates protein-protein interactions (1, 2). ANK proteins are among the most common structural motifs in eukaryotic cells and have a wide range of functions, including cytoskeletal regulation, transcriptional activation, and signaling transduction. Well-studied examples of ANK proteins include the members of the Ankyrin family, which connect integral membrane proteins to the spectrin-actin cytoskeleton (3) or mediate the regulation of the nuclear factor κ-light-chain enhancer of activated B cells (NF-κB) by the inhibitor of kB alpha (IκB-α), an ANK protein that binds and retains NF-κB in the cell cytosol in an inactive form (4). Although uncommon in viruses, ANK proteins are abundant in poxviruses. Between 30 and 50 ANK proteins exist in fowlpox or canarypox viruses, and up to 15 ANK orthologue groups can be found in cowpox virus (CPXV), most of which are conserved across mammal-infecting poxviruses, including variola virus (VARV) (5). A notable exception is molluscum contagiosum virus (MCV), a human-infecting poxvirus that lacks all ANK proteins (6). The origin of the poxviral ANK proteins is unknown, but they are believed to have been acquired as a consequence of one or several horizontal gene transfer events from eukaryotic hosts and to have been subsequently duplicated (5, 7).

Poxviruses are large double-stranded DNA (dsDNA) viruses infecting a wide spectrum of species ranging from insects to humans. Poxviruses infecting vertebrates are grouped into the subfamily *Chordopoxvirinae* and are currently classified into 10 different genera. Human pathogens such as VARV, the causative agent of smallpox, and vaccinia virus (VACV), the vaccine used to eradicate smallpox, belong to the genus *Orthopoxvirus* (OPV), which also includes animal pathogens with zoonotic potential such as CPXV and monkeypox virus (MPXV) or with a very restricted host range such as ectromelia virus (ECTV) and camelpox virus (CMLV). Approximately 50% of the genes identified in all sequenced chordopox viruses are conserved and locate in the central region of the linear genome, whereas the remaining 50% are genus or species specific and encode factors manipulating host responses (8, 9). Many of these modulatory factors are dedicated to host immune evasion, in particular, to the suppression of innate immune responses mediated by interferon (IFN) responsive factors (IRFs) and NF-κB and hence of the production of IFN and other proinflammatory mediators that induce the antiviral state (10). ANK proteins are commonly found on the genome termini, and, in agreement with their genomic location, some have been shown to modulate host range and cellular immune signaling. For instance, VACV protein K1 is necessary for VACV replication in various mammalian cell lines (11–16) and has been reported to modulate the activation of protein kinase R (17, 18). More recently, VARV protein G1 and its orthologues ECTV 002, CPXV 006, and MPXV 003 were shown to interact with the NF-κB precursor p105 and to block NF-κB activation (19–22).

A unique feature of some poxviral ANK proteins is their ability to associate with a Cullin-RING ubiquitin E3 ligase (CRL) containing S-phase-associated protein kinase 1 (Skp-1) and Cullin-1 (also known as SCF1 [Skp-1, Cul-1, F-box]) (23). In cells, CRL-1 complexes target cellular proteins for ubiquitylation by the use of F-box-containing proteins (24), which associate with Skp-1 and Cul-1 via an N-terminal F-box domain and recognize substrates via a second, independent protein-binding domain (25). Viral ANK proteins contain a C-terminal motif that resembles cellular F-box domains and mediates interaction with Skp-1 (26). The poxviral F-box (also known as the PRANC domain) is thereby proposed to hijack the cellular CRL-1 complex and redirect its ubiquitin targeting activity toward cellular substrates recognized by the ANK domain in a manner that is beneficial for the virus. Although the identity of these cellular substrates remains elusive in most cases, multiple poxviral ANK proteins have been experimentally shown to interact with CRL-1 (19, 22, 23) or to contain predicted F-box domains (7, 27). As a consequence, viral ANK proteins are structurally divided in ANK/F-box proteins and ANK-only proteins.

The origin and function of the majority of poxviral ANK proteins remain unknown. Here we combined computational analysis and unbiased quantitative proteomics to discover a new class of viral ANK proteins termed ANK/BC. ANK/BC proteins combine ANK domains with a C-terminal BC box to target the host E3 ligase Cullin-2 (Cul-2). We demonstrate that these viral ANK/BC proteins are potent immunomodulatory proteins inhibiting innate immune responses via the suppression of NF-κB and IRF-3 activation. Viral ANK/BC proteins targeting Cul-2-based CRLs represent a novel viral strategy to prevent innate immune activation and shed light onto the origin of the poxviral ANK protein family.

## RESULTS

### ECTV protein 010 is a noncanonical ANK protein that binds Cul-2.

**FIG 1:**
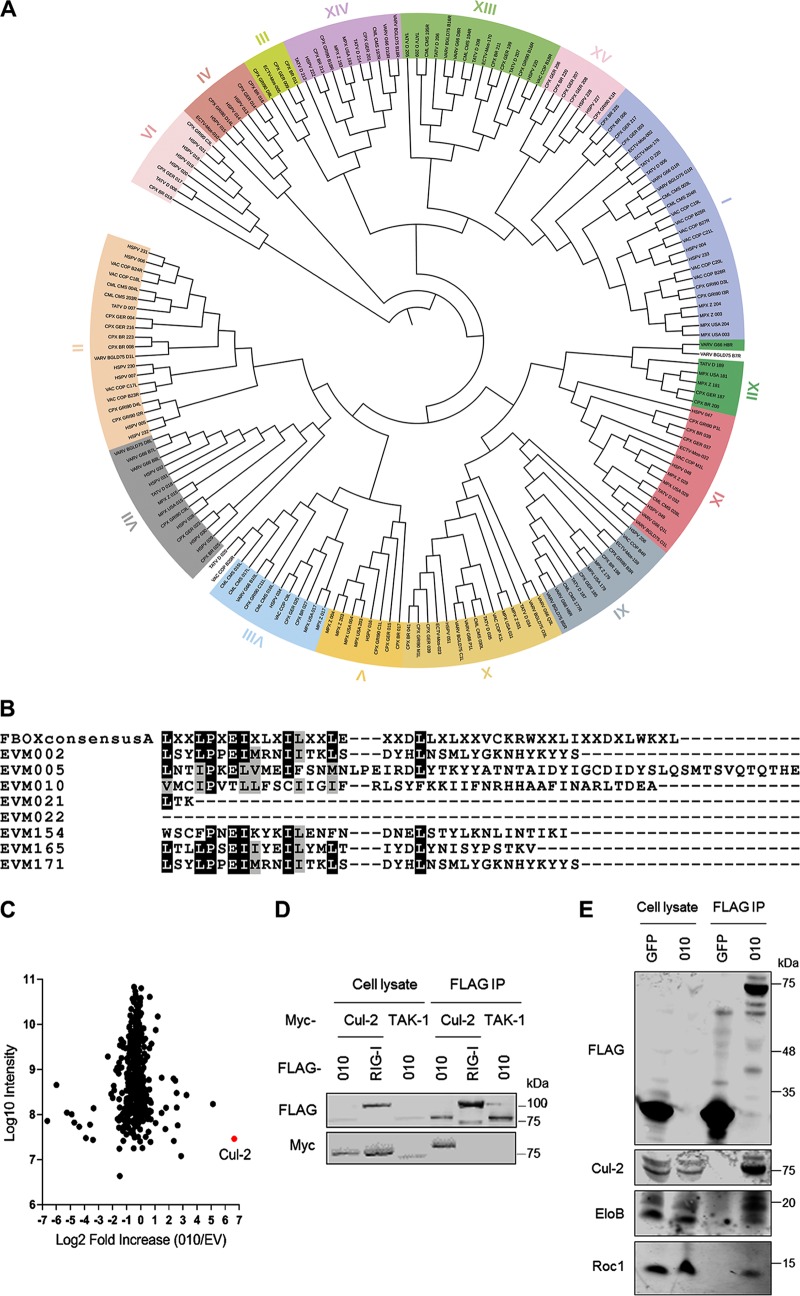
ECTV protein 010 is a noncanonical ANK protein that binds Cul-2. (A) Phylogenetic tree of OPV ANK proteins. Orthologue groups are indicated in roman numerals and highlighted in several colors. Protein designations without background color are those that could not be assigned to an orthologue group unambiguously. Branch length indicates relationship. (B) Alignment of the C-terminal terminations of ECTV ANK proteins in comparison to the consensus poxviral F-box sequence (top). Identical residues are highlighted in black. Similar residues are highlighted in gray. (C) SILAC proteomics analysis of FLAG-immunoprecipitated proteins from cells expressing FLAG-tagged 010 or the corresponding empty vector (EV). The mean log2 fold increase (010/EV) in expression of each protein identified in three biological replicates is presented in comparison to values representing the mean intensity levels of those proteins. The data point corresponding to Cul-2 is highlighted in red. (D) HEK293T cells were cotransfected with FLAG-tagged 010 or RIG-I together with myc-tagged Cul-2 or TAK-1. After 24 h, the cells were lysed and subjected to FLAG immunoprecipitation (IP). Cell lysates (2%) and IP samples were analyzed by immunoblotting against the indicated proteins. (E) HEK293T cells were transfected with FLAG-tagged 010 or GFP. After 24 h, the cells were lysed and subjected to FLAG IP. Cell lysates (1.25%) and IP samples were analyzed by immunoblotting against the indicated proteins.

To assess the relationships among poxviral ANK proteins, we initially retrieved the 181 predicted ANK proteins from 13 species belonging to the OPV genus and established their phylogenetic distribution (Fig. 1A). We identified 15 different orthologue groups as previously proposed (27) but also noticed that groups IV and VI clustered together separately from the other groups, indicating a closer relationship between these two groups relative to others. Group IV included ECTV protein 010 and CPXV protein 016 amongst others, whereas group VI included CPXV protein 019. Previous work from others identified a family of ANK/F-box proteins in ECTV that regulate NF-κB (22, 28, 29). ECTV 010 was not included in that family, however. To identify potential differences between 010 and the ANK/F-box proteins, we compared the sequences of all 8 predicted ANK proteins in ECTV. Alignment of the C-terminal tails extending from the ANK domains of these proteins identified the consensus sequence ϕ-Pro-X_2_-ϕ-X_3_-ϕ (where ϕ represents a hydrophobic amino acid and X represents any amino acid) of the F-box domain in the proteins 002/171, 005, 154, and 165 (Fig. 1B). Proteins 021 and 022 did not have any C-terminal extension since these are ANK-only proteins. Interestingly, protein 010 did have a C-terminal tail of a length similar to those observed in ANK-F-box proteins, but its sequence did not conform to the consensus F-box. The absence of a functional F-box domain was confirmed by the inability of 010 to pull down Cul-1 by coimmunoprecipitation (data not shown).

To gain insights into the biology of this noncanonical ANK protein, we performed unbiased quantitative proteomics on 010 using stable isotope labeling of amino acids in cell culture (SILAC). Isotope-labeled HEK293T cells were transfected with FLAG-tagged 010 or the corresponding empty vector (EV) in triplicate and subjected to FLAG immunoprecipitation 24 h later. Eluates were combined and analyzed by mass spectrometry (MS) to identify potential interacting partners of 010. Among the >500 proteins reliably identified, very few were significantly enriched in the 010 pulldown compared to the EV condition (see Data Set S1 in the supplemental material). Among these, the E3 ubiquitin ligase Cul-2 showed the highest fold increase (Fig. 1C). We did not detect traces of Cul-1 or any other Cul protein in association with 010. To test whether 010 interacted with Cul-2, we performed FLAG immunoprecipitation upon transfection of FLAG-010 and myc-Cul-2. Despite the low expression levels observed for 010, anti-myc immunoblotting revealed an interaction between 010 and Cul-2 that did not occur with other proteins containing the same tags (Fig. 1D). Furthermore, 010 interacted with endogenous levels of Cul-2 as well as with other components of Cul-2 complexes such as Elongin B and Roc1 (Fig. 1E).

### ECTV 010 targets Cul-2 via a C-terminal BC box.

**FIG 2:**
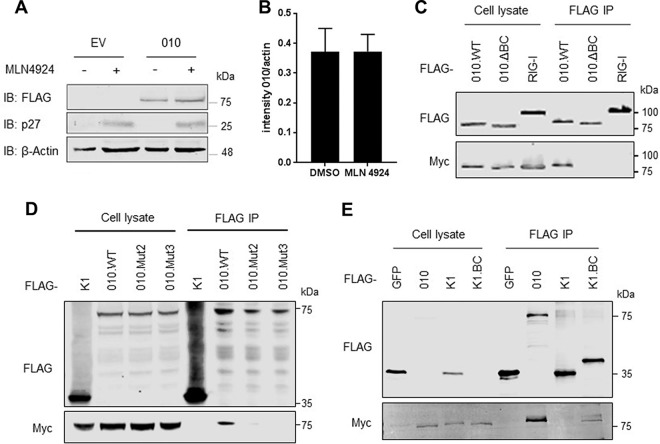
ECTV 010 targets Cullin-2 via a C-terminal BC box. (A) HEK293T cells were transfected with FLAG-tagged 010 or EV. After 24 h, the cells were treated with 1 μM MLN4924 or vehicle (DMSO) for a further 16 h. Cell lysates were subjected to immunoblotting (IB) against the indicated proteins. (B) Normalized intensity for 010 protein bands after DMSO or MLN4924 treatment from 3 independent experiments obtained via Li-COR. (C) HEK293T cells were cotransfected with FLAG-tagged full-length 010 (WT), 010 lacking the last 36 C-terminal amino acids (ΔBC), or RIG-I together with myc-tagged Cul-2. After 24 h, the cells were lysed and subjected to FLAG immunoprecipitation (IP). Cell lysates (2%) and IP samples were analyzed by immunoblotting against the indicated proteins. (D) HEK293T cells were transfected with FLAG-tagged full-length 010 (WT), 010.L728A/C723F (mut2), 010.L728A/C723F/I736A (mut 3), or the ANK-only protein K1 together with myc-tagged Cul-2. After 24 h, the cells were lysed and subjected to FLAG IP and immunoblotting as described above. (E) HEK293T cells were transfected with FLAG-tagged 010, GFP, K1, or K1 fused to the last 37 C-terminal amino acids of 010, together with myc-tagged Cul-2. After 24 h, the cells were lysed and subjected to FLAG IP and immunoblotting as described above. Data are representative of results obtained from at least 3 biological experiments.

Given the nature of Cul-2 as a ubiquitin ligase, we first addressed whether the 010-Cul-2 interaction would render 010 susceptible for proteasomal degradation. Cells were transfected with 010 or EV and subsequently treated with the neddylation inhibitor MLN4924, a drug that blocks the action of Cullins (30), or vehicle. Treatment with MLN4924 induced accumulation of p27, a known Cullin target, but did not affect the levels of 010 (Fig. 2A). Thus, ratios between 010 and β-actin remained similar in dimethyl sulfoxide (DMSO)- and MLN4924-treated cells (Fig. 2B). We then hypothesized that 010 could act as a Cul-2 adaptor. The best-studied cellular adaptor of Cul-2 is the Von Hippel-Lindau (VHL) protein, which recruits CRL-2 complexes to degrade hypoxia-inducible factors (31). VHL interacts with Cul-2 via a C-terminal domain known as the Elongin BC box followed by a Cul-2 box (32). A detailed analysis of the 010 amino acid sequence identified a consensus BC box ([T, S, P]-L-X_3_-[C, S, A]-X_3_-φ) at the C terminus (positions 727 to 736), the same region that proved dissimilar to the F-box domain. To determine whether this region contributed to the ability of 010 to bind Cul-2, a truncation was first generated in which the last 36 amino acids of 010 (including the BC box) were deleted. This construct (ΔBC) was expressed at levels similar to those seen with wild-type (WT) 010 but failed to interact with Cul-2 after immunoprecipitation (Fig. 2C). We then generated a construct in which the critical Leu, Cys, and Ile residues of the cryptic BC motif were mutated (L728A/C723F [named “mut2”] and L728A/C723F/I736A [named “mut3”]). Both alleles were expressed to the same extent as the ΔBC and WT 010 alleles and failed to coprecipitate with Cul-2, indicating that mutagenesis of Leu and Cys in the BC box is sufficient to abolish interaction with Cul-2 (Fig. 2D). We next tested whether the presence of the BC box of 010 could mediate association with Cul-2 in other viral ANK proteins. The BC box-containing tail of 010 was fused to the C terminus of VACV protein K1, an ANK-only protein containing 9 ANK repeats (33). As expected, K1 did not copurify with Cul-2, but the K1.BC chimera did, despite the lower expression levels observed for this fusion protein (Fig. 2E). Taken together, these results demonstrated that 010 is a noncanonical ANK protein that binds to Cul-2 complexes using a C-terminal cryptic BC box.

### ANK/BC proteins are conserved among several OPV.

**FIG 3:**
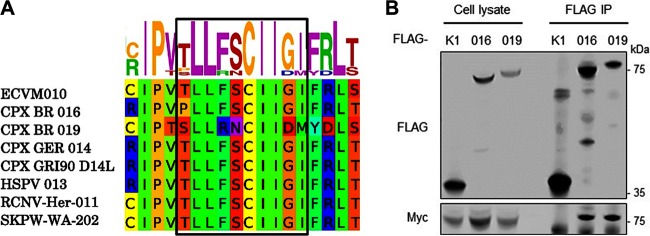
ANK/BC proteins are conserved among several OPV. (A) Conservation of the BC box sequence in the indicated ANK proteins. (B) HEK293T cells were cotransfected with FLAG-tagged 016, 019, or K1 together with myc-tagged Cul-2. After 24 h, the cells were lysed and subjected to FLAG immunoprecipitation (IP). Cell lysates (2%) and IP samples were analyzed by immunoblotting against the indicated proteins.

Next we established whether targeting host Cul-2 was a strategy unique to ECTV or was shared by other OPV. We initially performed a bioinformatics search for ANK proteins containing a BC box pattern in the last third of the protein (the C terminus) as discovered in ECTV 010. Several proteins, including CPXV 016, CPXV 019, horsepox virus 013, raccoonpox virus 011, and skunkpox virus 202, were identified (Fig. 3A). Most of these proteins belonged to ANK orthologue groups IV and VI (Fig. 1A). We then exploited SILAC proteomics to identify viral Cul-2 interacting proteins during infection. Full-length Cul-2 and its dominant-negative N-terminal domain (Cul-2.NTD) tagged with a C-terminal tandem affinity purification (TAP) tag containing FLAG epitopes were transfected into isotopically labeled HEK293T cells and subsequently infected for a further 16 h with 2 PFU per cell of CPXV strain Brighton Red. Mass spectrometry analysis of the FLAG immunoprecipitates identified a significant enrichment of viral ANK proteins CPXV 016 (the homologue of ECTV 010) and CPXV 019 in the Cul-2 and Cul-2.NTD pulldowns compared to the EV condition (Data Set S2), demonstrating that these proteins interact with Cul-2 during virus infection. No other viral proteins were significantly enriched. To confirm that these proteins interacted with Cul-2, their codon-optimized sequences were cloned and overexpressed in HEK293T cells together with myc-Cul-2. Both proteins coprecipitated with Cul-2 under conditions in which the ANK-only protein K1 did not (Fig. 3B). Taken together, these data reveal that poxviral ANK orthologue groups IV and VI represent a novel class of viral ANK proteins targeting host Cul-2.

### ANK/BC proteins are potent inhibitors of innate immune signaling.

**FIG 4:**
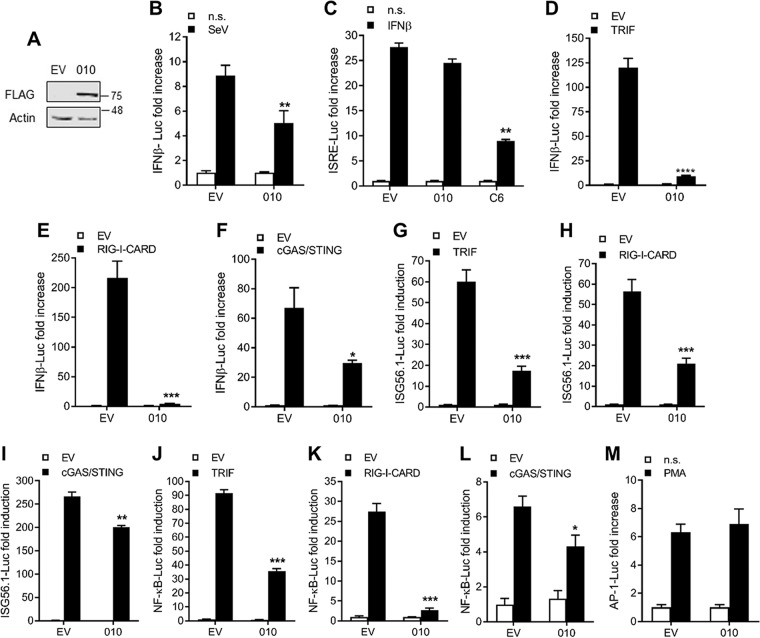
ECTV 010 inhibits NF-kB and IRF-3 activation downstream of multiple PRRs. (A) Immunoblot from a representative reporter assay in HEK293T cells transfected with 25 ng/well of 010 or the corresponding empty vector (EV). Lysates were cleared by centrifugation and subjected to immunoblotting against the indicated proteins. (B) Cells were transfected with 010 or EV together with 10 ng/well of pTK-*Renilla* and 70 ng/well of pIFN-β–Luc. After 24 h, the cells were stimulated with Sendai virus (SeV) for a further 24 h and luciferase activity was measured. Firefly luciferase (FLuc)/*Renilla* Luc ratios were normalized to the nonstimulated (n.s.) control and are presented as fold increase values. (C) Cells were transfected as described for panel B but with 70 ng/well of pISRE-Luc and including C6 as a positive control. The cells were stimulated 24 h after with 50 ng/ml of IFN-β for a further 8 h. (D to F) Cells were transfected as described for panel B together with (D) 30 ng/well of TRIF, (E) 5 ng/well of RIG-I-CARD, or (F) 20 ng/well each of cGAS and STING. (G to I) Cells were transfected as described for panels D to F but with 70 ng/well of pISG56.1-Luc. (J to L) Cells were transfected as described for panels D to F but with pNF-κB–Luc. (M) Cells were transfected as described for panel B but with 250 ng/well of pAP-1–Luc. For all assays, data are presented as means ± SD and represent results from one experiment that is representative of at least three, each performed in triplicate. *, *P* < 0.05; **, *P* < 0.01; ***, *P* < 0.001 (unpaired Student's *t* test).

We then aimed to ascribe a function to the newly identified ANK/BC proteins. ECTV protein 010 is an uncharacterized protein conserved across ECTV strains that is located close to the left genome terminus downstream of an early promoter element (34, 35). These features supported the idea of a potential immunomodulatory role for protein 010. To address this possibility, we first employed reporter gene assays in cells transfected with either 010 or EV (Fig. 4A). Expression of 010 reduced the activation of an IFN-β reporter upon infection with Sendai virus (SeV), a strong inducer of IFN-β production (Fig. 4B), but did not affect the activation of the antiviral signaling mediated by the Janus kinase/signal transducers and activators of transcription (JAK/STAT) pathway in response to IFN-β (Fig. 4C). 010 also blocked activation of the IFN-β reporter triggered by the overexpression of the Toll–interleukin-1 receptor (TIR) adaptor TRIF (Fig. 4D), the RNA sensor RIG-I (Fig. 4E), and the cytosolic DNA sensors cGAS and STING (Fig. 4F). We further mapped the inhibitory action of 010 using luciferase (Luc) reporters specific for IRF-3, NF-κB, or mitogen-activated protein kinase (MAPK)/activator protein 1 (AP-1), the 3 transcription factors coordinating optimal type I IFN responses. Expression of 010 blocked IRF-3 signaling downstream of TRIF (Fig. 4G), RIG-I (Fig. 4H), and, to a lesser extent, cGAS/STING (Fig. 4I). Likewise, activation of NF-κB by TRIF (Fig. 4J), RIG-I (Fig. 4K), or cGAS/STING (Fig. 4L) was also significantly reduced by expression of 010. In contrast, no effect was observed on the activation of the MAPK/AP-1 reporter upon treatment with phorbol 12-myristate 13-acetate (PMA) (Fig. 4M).

**FIG 5:**
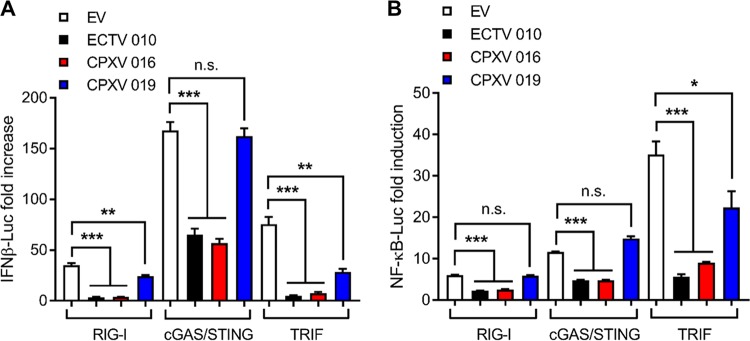
CPXV 016 and 019 inhibit NF-κB and IRF-3 activation. HEK293T cells were transfected with 25 ng/well of 010 (black bars), 016 (red bars), 019 (black bars), or the corresponding empty vector (EV; white bars) together with 10 ng/well of pTK-*Renilla* and 70 ng/well of (A) pIFN-β–Luc or (B) pNF-κB–Luc, as well as 50 ng/well of RIG-I, 20 ng/well each of cGAS and STING, or 10 ng/well of TRIF. After 16 h, the cells were harvested and luciferase activity was measured. FLuc/*Renilla* Luc ratios were normalized to the nonstimulated EV control and are presented as fold increase values. In all assays, data are presented as means ± SD and represent results from one experiment that is representative of at least three, each performed in triplicate. n.s. nonsignificant; *, *P* < 0.05; **, *P* < 0.01; ***, *P* < 0.001 (unpaired Student's *t* test).

We also studied the ability of CPXV 016 and 019 to modulate innate immune signaling. Cells were transfected with IFN-β and NF-κB reporter constructs, and RNA sensing, DNA sensing, and Toll-like receptor (TLR) signaling pathways were stimulated by overexpression of RIG-I, cGAS/STING, and TRIF, respectively, in the presence or absence of CPXV 016 or CPXV 019. CPXV 016 inhibited IFN-β (Fig. 5A) and NF-κB (Fig. 5B) activation downstream of the multiple pattern recognition receptor (PRR) as was observed for ECTV 010, confirming the broad and potent inhibitory role of this viral protein. In contrast, CPXV 019 was able to block TLR signaling but failed to affect RNA and DNA sensing pathways (Fig. 5).

### ANK/BC proteins suppress the production of CXCL10, CCL5, and IFN.

**FIG 6:**
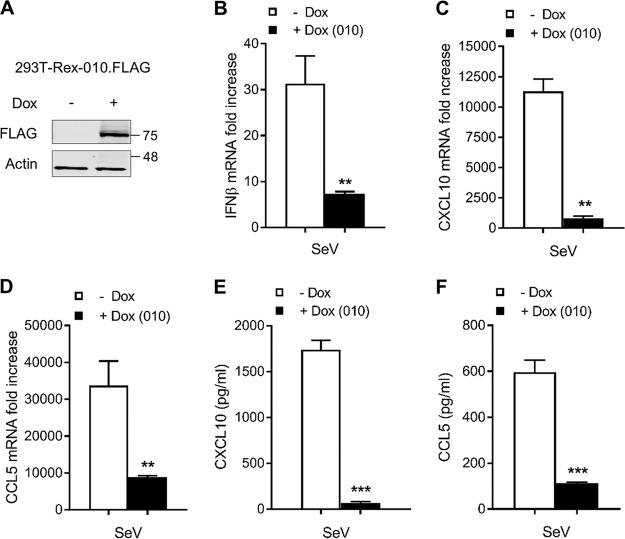
ANK/BC proteins suppress the production of CXCL10, CCL5, and IFN. (A) HEK293T-REx cells were induced with 2 μg/ml of doxycycline (Dox) for 16 h and subjected to immunoblotting against the indicated proteins. (B to D) The cells were induced with Dox as described for panel A and subsequently stimulated with SeV for a further 24 h. RNA expression levels for (B) IFN-β, (C) CXCL10, and (D) CCL5 were measured by quantitative PCR (qPCR). Data were normalized to 18S expression levels; data represent fold increase over the levels seen under nonstimulated conditions. (E and F) The cells were treated as described for panel B, and the resulting cell culture medium was subjected to ELISA to detect soluble levels of (E) CXCL10 and (F) CCL5. In all assays, data are presented as means ± SD and represent results from one experiment that is representative of at least three, each performed in triplicate. **, *P* < 0.01; ***, *P* < 0.001 (unpaired Student's *t* test).

To confirm the immunomodulatory capacity of ANK/BC proteins, quantitative PCR and enzyme-linked immunosorbent assays (ELISA) were performed. An inducible cell line expressing FLAG-010 upon addition of doxycycline (Dox) was generated (Fig. 6A). Cells were treated with Dox for 24 h and subsequently infected with SeV for a further 24 h before the expression levels of cytokines IFN-β, CXCL10, and CCL5 were measured by quantitative PCR. SeV infection induced an approximately 30-fold increase in IFN-β expression, and this was reduced to <10-fold by 010 in a statistically significant manner (Fig. 6B). Similarly, the expression levels of cytokines CXCL10 (Fig. 6C) and CCL5 (Fig. 6D) were also reduced in the presence of 010. Finally, levels of soluble CXCL10 and CCL5 were measured by ELISA. In agreement with mRNA quantitations, expression of 010 markedly reduced SeV-induced levels of CXCL10 and CCL5 (Fig. 6E and F). Collectively, these results demonstrate that ANK/BC proteins are potent immunomodulators suppressing the production of type I IFN and innate immune mediators downstream of multiple PRRs.

### Association with Cul-2 correlates with ANK/BC optimal inhibitory activity.

**FIG 7:**
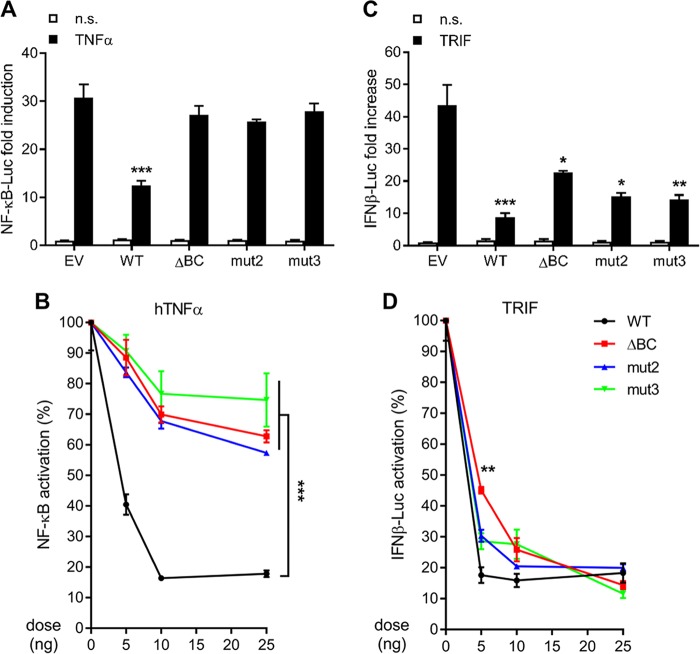
Association with Cul-2 is required for ANK/BC optimal inhibitory activity. (A) HEK293T cells were transfected with 5 ng/well of 010, 010dBC, 010.L728A/C723F (mut2), 010.L728A/C723F/I736A (mut 3), or the corresponding empty vector (EV) together with 10 ng/well of pTK-*Renilla* and 70 ng/well of pNF-κB–Luc. After 24 h, the cells were stimulated with TNF-α for a further 6 h. FLuc/*Renilla* Luc ratios were normalized to the nonstimulated (n.s.) EV control and are presented as fold increase values. (B) NF-κB activation was assessed as described above but in the presence of increasing doses of each 010 construct. Luciferase ratios were plotted as percentages relative to the extent of NF-κB activation observed in EV-transfected cells stimulated with TNF-α. (C) Cells were transfected as described for panel A but together with 20 ng/well of TRIF. After 16 h, the cells were harvested and luciferase activity was measured. FLuc/*Renilla* Luc ratios were normalized to the nonstimulated (n.s.) EV control and are presented as fold increase values. (D) IFN-β activation was assessed as described above but in the presence of increasing doses of each 010 construct. Luciferase ratios were plotted as percentages relative to the extent of IFN-β activation observed in EV-transfected cells stimulated with TRIF. In all assays, data are presented as means ± SD and represent results from one experiment that is representative of at least three, each performed in triplicate. *, *P* < 0.05; **, *P* < 0.01; ***, *P* < 0.001 (unpaired Student's *t* test).

Finally, we addressed the impact of the BC box on the ability of 010 to inhibit innate immunity. WT 010 and mutants of 010 unable to bind to Cul-2 were overexpressed, and their ability to block intracellular signaling was assessed. In contrast to the results seen with WT 010, 010 mutants ΔBC, mut2, and mut3 were significantly impaired in their ability to suppress NF-κB activation stimulated by the addition of tumor necrosis factor alpha (TNF-α) (Fig. 7A). We then assessed this inhibition at multiple doses of 010 and their mutants and confirmed that all constructs that were unable to bind Cul-2 were also unable to suppress NF-κB activation irrespective of the dose tested (Fig. 7B). A similar trend was observed when assessing IFN-β activation. While WT 010 significantly inhibited the activation of the IFN-β reporter after TRIF overexpression, the 010 ΔBC, mut2, and mut3 mutants showed more modest inhibitory capacity (Fig. 7C). In contrast to the NF-κB signaling results, all the mutants suppressed IFN-β activation as well as wild-type 010 at higher doses (Fig. 7D), highlighting the possibility of the presence of different mechanisms of inhibition of these signaling pathways. Similar results were obtained in stimulating the NF-κB reporter with interleukin-1β (IL-1β) and the IFN-β reporter with RIG-I overexpression (data not shown). The resulting data demonstrated that optimal suppression of innate immune signaling by 010 requires the presence of an intact C-terminal BC box, strongly suggesting that interaction with host Cul-2 is needed for this function. Collectively, these results reveal for the first time the poxviral targeting of Cul-2 via molecular mimicry of a cellular Cul-2 interacting motif to antagonize antiviral signaling and IFN-β production.

## DISCUSSION

The ANK motif is among the most abundant in nature, particularly in eukaryotes, where it is typically involved in protein-protein interactions. ANK proteins also constitute the largest family of poxvirus proteins, representing up to 15% of the virus proteome (5, 26, 36, 37). Most poxviral ANK proteins contain a conserved C-terminal domain homologous to the cellular F-box domain that is present in CRL-1 substrate adaptor proteins (23, 26). Cellular F-box proteins use the F-box sequence to recruit CRL-1 and direct its E3 ubiquitin ligase activity toward proteins recruited via an independent protein-protein interaction domain (25). Although very few substrates have been identified for poxvirus ANK/F-box proteins, it is believed that viral ANK/F-box proteins act in a manner similar to that seen with their cellular counterparts rather than targeting and inhibiting CRL-1 generically (37). In addition, poxviruses express ANK-only proteins that do not contain F-box domains, but it is unclear whether these evolved from full-length ANK/F-box proteins or were acquired independently (7, 26). Our results demonstrate that a third class of ANK proteins exists in poxviruses, namely, ANK/BC proteins. These proteins do not interact with cellular CRL-1 but rather with CRL-2 complexes. This interaction is mediated by a motif in their C terminus that mimics the Elongin BC box present in cellular Cul-2 adaptors such as protein VHL. Mutagenesis of key residues in this motif abolishes binding to Cul-2, whereas its fusion to an ANK-only protein is sufficient to mediate such interactions. The crystal structure of the VHL:Elongin B/C complex has shown that residues Leu158 and Cys162 in VHL are critical in mediating binding, whereas the third conserved residue in the BC motif (Val166) provides further hydrophobic interactions (38). This model seems to apply to viral ANK/BC proteins, given that replacement of Leu and Cys residues was sufficient to abrogate binding. Cellular proteins containing BC boxes can interact with Cul-2 or Cul-5, and this has been shown to be specified by a second motif located downstream of the BC box that contains a critical Pro and several hydrophobic residues (32, 39). In the case of viral ANK/BC proteins, no Pro is found downstream of the BC motif and there are no sequences with homology to Cul-2 or Cul-5 boxes. The entire domain downstream of the BC motif is shorter than in cellular Cul-2 interacting proteins, and there is limited sequence homology to them. Similarly to the viral F box (40), it appears that the viral BC box is truncated and has retained only the most critical residues allowing Cul-2 interaction. This lack of conservation may have hindered its discovery and suggests the existence of more BC motifs than previously inventoried.

Despite its presence in all lineages of chordopox viruses, the origin of the poxvirus ANK proteins remains unclear. The absence of a cellular protein combining ANK with an F-box domain precludes the possibility of a direct acquisition event by an ancestor virus. However, cellular ANK-containing proteins that interact with CRL machinery via a C-terminal adaptor domain do exist. Suppressor of cytokine signalling (SOCS) proteins interact with CRL-5 and inhibit the JAK/STAT signaling pathway (41–43). Also, FEM1 proteins interact with CRL-2 to modulate transcriptional activity (44, 45). The discovery of viral ANK/BC proteins in poxviruses suggests an evolutionary model in which an ancestral ANK/BC gene was acquired by an ancestor virus common to all lineages of chordopox viruses and was subsequently duplicated and adapted to new hosts and functions such as targeting CRL-1 complexes. This ancient gene may have been lost during the development of the molluscipoxvirus lineage, explaining the absence of ANK proteins in MCV. In addition, viral ANK/BC proteins correspond to OPV orthologue groups IV and VI, which cluster separately in phylogenetic analysis and are unique to CPXV and ECTV. The presence of ANK/BC proteins in CPXV—the OPV with the largest set of ANK proteins—is in line with studies suggesting that this virus is more closely related to the OPV ancestor (8) and that adaptation to new hosts involved gene loss from a CPXV-like ancestor (46). Therefore, CPXV ANK/BC genes may represent remnants of the original ANK/BC gene that were lost in other viral groups through adaptation.

Poxviruses dedicate a large portion of their coding capacity to antagonizing host innate immunity. Their antagonism of NF-κB signaling, for instance, is remarkable and highlights the importance of this transcription factor in the biology of the virus (10, 47, 48). NF-κB activation relies on the degradation of the inhibitor of κB (IκB)-α by a CRL-1 complex containing the E3 ligase β-TrCP (49). β-TrCP is one of the 69 F-box proteins predicted to exist in mammalian cells (24, 50). It binds IκBα through a WD40 domain and recruits the CRL-1 machinery via the F-box domain, presumably in competition with other cellular F-box proteins (25, 51, 52). It is thus reasonable to envisage that adaptation of the BC box of an original ANK/BC protein into an F-box could have been evolutionarily favored. This new ANK/F-box combination would have allowed the virus to hijack the cellular CRL-1 machinery and, in doing so, to compete with β-TrCP and prevent IκBα degradation and NF-κB activation. The critical role of β-TrCP action in poxvirus infection is highlighted by the existence of a viral Bcl-2-like protein that specifically blocks its ability to recognize and degrade substrates (53–55). Similarly, the advantage of hijacking CRL-1 is demonstrated by the conservation of the F-box domain during the expansion of the ANK/F-box family. This expansion presumably increased the repertoire of cellular substrates recruited to virally assembled CRL-1 complexes. Interestingly, ANK/BC proteins were also potent suppressors of IRF-3 responses. In contrast to NF-κB inhibition, this function did not require an intact BC box because both the ΔBC mutant and the point mutants retained considerable inhibitory activity. Thus, ANK/BC proteins may also contain domains that can function independently of CRL complex formation, a case that has also been described for some ANK/F-box proteins (56). It is also formally possible that the BC box mediates a novel and yet unknown function that is required for NF-κB suppression but not IRF-3 suppression. Taking the data together, ANK/F-box and ANK/BC proteins provide evidence of the close interplay between viruses and the host CRL machinery. In the case of poxviruses, this is further emphasized by the presence of viral adaptors of CRL-3 (37). It remains unknown whether poxviruses utilize other CRLs, but a recent report demonstrated that MCV targets CRL-5 to degrade p65 (57). This suggests that noncanonical mechanisms for CRL manipulation may also exist.

Our results identify a new class of poxviral ANK proteins and demonstrate their potent antagonism of innate immune signaling. Optimal antagonism required interaction with Cul-2, presumably because the process of binding and degrading a substrate via CRL-2 machinery is more effective than simply binding the substrate. Both orthologue group IV and group VI proteins interacted efficiently with Cul-2, but the two groups showed different inhibitory capacities against multiple innate immunity signaling pathways, implying the existence of a third factor (presumably a substrate) unique to each viral adaptor protein. This substrate(s) would have likely been missed in our proteomics study due to rapid Cul-2-mediated degradation. Although the substrates of most poxviral ANK CRL adaptors remain unknown, our data demonstrate that, in the case of viral ANK/BC proteins, these must be components of the innate immune signaling cascades leading to or regulating IFN production. Identification of these components will be the subject of future studies. Taking the data together, we reveal for the first time the existence of viral ANK proteins adapted to manipulate CRL-2 and the existence of a novel mechanism used by poxviruses to antagonize the host innate immune response.

## MATERIALS AND METHODS

### Cells, viruses, and reagents.

HEK293T, BS-C-1, and RK-13 were grown in Dulbecco modified Eagle medium (DMEM) (Life Technologies) supplemented with 10% heat-inactivated fetal calf serum (FCS) (Seralab), 100 U/ml penicillin, and 100 μg/ml streptomycin (Pen/Strep) (Life Technologies). HEK293T-REx cells were grown as described above with the addition of 10 μg/ml blasticidin (Life Technologies). ECTV strain Moscow was from Antonio Alcami and was expanded in BS-C-1 cells. CPXV strain Brighton Red was from Geoffrey L. Smith and was expanded in RK-13 cells. Both viruses were titrated by conventional plaque assay. SeV was a gift from Steve Goodbourn. IL-1β, TNF-α, and IFN-β were from Peprotech. PMA was from Santa Cruz Biotechnology. MLN4924 was from Cayman Chemical.

### Expression vectors.

Gene sequences for ECTV 010, VACV K1, CPXV 016, and CPXV 019 were optimized for their expression in human cells and purchased from GeneArt (Life Technologies). Genes were subcloned into a pcDNA4/TO expression vector (Invitrogen) previously modified to express genes in frame with three C-terminal copies of the FLAG epitope in tandem. ECTV 010.ΔBC was obtained by PCR amplification of the gene fragment encompassing residues 1 to 726. ECTV 010 mutants L728A/C723F and L728A/C723F/I736A were obtained by site-directed mutagenesis using KOD Hot-start DNA polymerase (Merck-Millipore). Fusion of the 37 ECTV 010 C-terminal amino acids to VACV K1 was performed by overlapping PCR, and the product was subsequently cloned into pcDNA4/TO-3xFLAG. Myc-tagged human Cul-2 was from Wenyi Wei (58). Cul-2.TAP and Cul-2.NTD.TAP were generated by PCR amplification of full-length Cul-2 and of the region encompassing amino acids 1 to 454, respectively. PCR amplicons were subsequently ligated into pcDNA4/TO in frame with a C-terminal TAP tag containing 2 copies of the Strep tag and 1 copy of the FLAG tag as previously described (59, 60). FLAG-tagged RIG-I and TAP-tagged cGAS were generated by PCR amplification using cDNA from THP-1 cells. FLAG-tagged RIG-I.CARD was from Adolfo Garcia-Sastre. FLAG-tagged STING was from Brian J. Ferguson. FLAG-tagged green fluorescent protein (GFP) and FLAG-tagged TRIFΔRIP (here referred to as TRIF) were from Felix Randow. All constructs were verified by sequencing.

### Phylogenetic studies.

A total of 181 ANK protein sequences from OPV were fetched from the Viral Orthologous Clusters (https://virology.uvic.ca/). Each protein was assigned to a previously described orthology group (27). An individual multiple-sequence alignment (MSA) was done for every orthologue group; subsequently, the MSA data were merged by the use of makemergetable from MAFFT. A phylogenetic tree was obtained with RAxML (raxmlHPC -T 4 -m PROTCATAUTO -p 12367 -x 12367 -#100 -f a) (61) after checking for good-quality alignment with Gblocks (62). ANK proteins were predicted with hmmscan (HMMER 3.1b2; http://hmmer.org/) against the PFAM database (63), retaining just those with an E value of <0.01 and an I value of <0.01. BC box pattern searches {in prosite format as follows: [TSP]-L-x(3)-[CSA]-x(3)-[AILMFVPG]} were performed for all the proteins with emboss fuzzpro (64). Protein sequences with ANK domains and a BC box in the last third of the protein sequence were selected. Protein sequence terminations were aligned using MAFFT (65).

### SILAC quantitative proteomics.

HEK293T cells were cultured at least 5 times in Arg/Lys-free minimal essential medium (MEM) supplemented with Pen/Strep, dialyzed FCS, and either unlabeled or stable isotope-labeled forms of Arg and Lys (DC Biosciences). Cells were transfected with 10 μg of the indicated plasmids by the use of polyethylenimine (PEI; Polysciences), and lysates were subjected to FLAG immunoprecipitation as described below after total protein normalization was performed using a bicinchoninic acid protein assay (Pierce). Denatured eluates were combined at a 1:1 ratio and subjected to in-gel tryptic digestion using a ProGest automated digestion unit (Digilab). The resulting peptides were fractionated using an Ultimate 3000 nanoHPLC system in line with an Orbitrap Fusion Tribrid mass spectrometer (Thermo Scientific). All spectra were acquired using Xcalibur 2.1 software (Thermo Scientific) and operated in data-dependent acquisition mode. FTMS1 spectra were collected at a resolution of 120,000 over a scan range (*m*/*z*) of 350 to 1,550, with an automatic gain control (AGC) target of 300,000 and a maximum injection time of 100 ms. Precursors were filtered using an intensity range of 1E−4 to 1E−20 and in accordance with the charge state (to include charge states 2 to 6) and with monoisotopic precursor selection. Previously interrogated precursors were excluded by the use of a dynamic window (40 s, ±10 ppm). The MS2 precursors were isolated with a quadrupole mass filter set to a width of 1.4 *m*/*z*. ITMS2 spectra were collected with an AGC target of 20,000, a maximum injection time of 40 ms, and a collision-induced dissociation (CID) collision energy level of 35%.

### Mass spectrometry data analysis.

The raw data files were processed and quantified using Proteome Discoverer software v1.4 (Thermo Scientific) and searched against the UniProt human database (downloaded 14 September 2017; 140,000 entries) plus the ECTV 010 protein sequence or the entire CPXV proteome sequence using the SEQUEST algorithm. Peptide precursor mass tolerance was set at 10 ppm, and the tandem MS (MS/MS) tolerance value was set at 0.6 Da. Search criteria included carbamidomethylation of cysteine (+57.0214 Da) as a fixed modification and oxidation of methionine (+15.9949 Da) and SILAC labels (+6.02 Da [R] or +10.008 Da [R] and +4.025 Da [K] or +8.014 Da [K]) as variable modifications. Searches were performed with full tryptic digestion, and a maximum of 1 missed cleavage was allowed. The reverse database search option was enabled, and all peptide data were filtered to satisfy the criterion of a 1% false-discovery rate. Contaminants, reverse database hits, and hits corresponding to a single peptide were removed. Protein ratios were calculated and converted into the corresponding log2 values. Putative interaction partners were selected when their ratios were above the cutoff value (mean + 1.96 standard deviation [SD]) and had been identified in at least two of the three replicates unless otherwise indicated.

### Immunoprecipitation.

HEK293T cells were seeded in 10-cm-diameter dishes and transfected with 5 μg of the indicated plasmids using PEI. After 24 h cells, were lysed with phosphate-buffered saline (PBS) supplemented with 0.5% NP-40 and protease and phosphatase inhibitors (Roche). Cleared lysates were incubated with FLAG M2 resin (Sigma) for 16 h at 4°C. For the analysis of Cul-2 endogenous complexes, the cells were lysed in 50 mM Tris-HCl (pH 7.5)–250 mM NaCl–1% NP-40–1 mM EDTA–1 mM dithiothreitol (DTT). The next day, the beads were washed 3 times with lysis buffer prior to incubation at 95°C for 5 min in Laemmli loading buffer to elute bound proteins. Cleared lysates and FLAG eluates were analyzed by SDS-PAGE and immunoblotting. Data shown are representative of at least 3 independent experiments showing similar results.

### SDS-PAGE and immunoblotting.

Samples were resolved by SDS-PAGE and transferred to nitrocellulose membranes (GE Healthcare) using a Trans-Blot semidry transfer unit (Bio-Rad). Membranes were blocked in 0.1% Tween–PBS supplemented with 5% skimmed milk (Sigma) and subjected to immunoblotting with the following primary antibodies at the indicated dilutions: FLAG (Sigma) (1:20,000), Myc (Merck-Millipore) (1:2,000), Cul-2 (Abcam) (1:1,000), Elongin B (Santa Cruz Biotechnology) (1:200), Roc-1 (Cell Signaling) (1:1,000), p27 (Cell Signaling) (1:1,000), α-tubulin (Upstate Biotech) (1:10,000), and β-actin (Sigma) (1:2,000). Primary antibodies were detected using IRDye-conjugated secondary antibodies in an Odyssey Infrared Imager (Li-COR Biosciences). Images were analyzed using Odyssey software, and quantitative data were obtained after integration of at least 3 independent experiments.

### Reporter gene assays.

HEK293T cells were seeded in 96-well plates and transfected with the indicated reporters and expression vectors using PEI as described in the figure legends. The reporter plasmids have been described previously (54). After 24 h, the cells were stimulated by infection with SeV for a further 24 h or by addition of 50 ng/ml of TNF-α for 6 h or of 50 ng/ml of IFN-β for 8 h or of 10 ng/ml of PMA for 16 h or by cotransfection of 30 ng/well of TRIF or 20 ng/well of cGAS and STING or 5 ng/well of RIG-I.CARD or 50 ng/well of RIG-I unless otherwise indicated. After stimulation was performed, cells were washed with ice-cold PBS and lysed with passive lysis buffer (Promega). Luciferase activity was measured in a Clariostar plate reader (BMG Biotech), and firefly and *Renilla* ratios were calculated under each set of conditions. Data were normalized to mock-infected samples or samples transfected with an empty vector and are presented as fold increase values.

### ECTV 010 inducible cell line.

HEK293T-REx cells were transfected with 010.FLAG using PEI and were subjected to endpoint limiting dilution in medium containing 10 μg/ml of zeocin (Life Technologies). After approximately 10 days, individual clones were selected and expanded. 010 expression was tested by immunoblotting upon addition of 2 μg/ml of doxycycline (Dox; Sigma). Clones showing the highest Dox-dependent expression levels were further selected.

### Quantitative PCR.

RNA was purified using a total RNA purification kit (Norgen Biotech). A 1-μl volume of RNA was transformed into cDNA using Superscript III reverse transcriptase (Invitrogen). cDNA was diluted 1:5 in water and used as a template for real-time PCR using SYBR Green PCR master mix (Applied Biosystems) in a LightCycler 96 system (Roche). Expression of each gene was normalized to an internal control (18S) gene, and these values were then normalized to the nonstimulated mock-infected control cells to yield a fold induction value. Primers used for the detection of CXCL10 (66), CCL5 (54), human IFN-β (hIFN-β) (67), and 18S (67) have been described previously.

### ELISA.

Cell culture supernatants from virus-infected HEK293T cells grown in 6-well plates were assayed for CXCL10 and CCL5 using Duoset ELISA reagents (R&D Biosystems) according to the manufacturer’s instructions.

### Statistical analysis.

Statistical significance was determined using a Student's pairwise *t* test, comparing each experimental condition to the control condition, with Welch’s correction where appropriate.

## Supplementary Material

Supplemental file 1

Supplemental file 2
